# Factors Influencing Quality of Life in Caregivers of People with Parkinson's Disease and Implications for Clinical Guidelines

**DOI:** 10.1155/2012/190901

**Published:** 2012-12-20

**Authors:** D. Morley, S. Dummett, M. Peters, L. Kelly, P. Hewitson, J. Dawson, R. Fitzpatrick, C. Jenkinson

**Affiliations:** Department of Public Health, University of Oxford, Old Road Campus, Headington, Oxford OX3 7LF, UK

## Abstract

The quality of life (QoL) of informal caregivers can be adversely affected by a number of factors. This issue, however, has not been well explored for carers of people with Parkinson's (PwP), with research largely restricted to the assessment of caregiver burden and caregiver strain. This study aims to determine the main influences on carer QoL in this population and consider results in the context of current clinical guidelines for the management of Parkinson's disease (PD). Carers completed the newly validated PDQ-Carer, and PwP completed the PDQ-39. The sample comprised 238 carers (mean age 68.20 years) and 238 PwP (mean age 71.64). Results suggest multiple influences on caregiver QoL. These include carer age, gender, health status, and duration of the caregiving role. PwP levels of mobility and cognitive impairment are also significant influences on carer QoL. Not only should practitioners and service providers be particularly aware of the heightened impact of PD on carers over time and as PwP symptoms deteriorate, but this should also be reflected in clinical guidelines for the management of PD.

## 1. Introduction

The savings to the United Kingdom (UK) government from the care provided by an estimated six million unpaid informal carers is substantial [1], with one estimate suggesting it may be as great as *£*119 billion annually [2]. The profile of such carers has been raised significantly in recent years as the pressures facing them are considerable; 77% report deterioration in health as a direct result of their caregiving role [3]. With an ageing population, such pressures are likely to increase, and it has been suggested that, by 2017, the demands on informal carers will be outweighed by what they can realistically provide [4]. Such a context highlights the importance of not only identifying the needs of carers, but also ensuring that these needs are reflected in guidance purporting to set the highest standards for healthcare and healthy living.

The features of a particular condition affecting a patient are likely, in part, to determine the impact on their carer [5]. Parkinson's disease (PD), for example, is a chronic progressive condition characterised by tremor, bradykinesia, and rigidity [6]. People with Parkinson's disease (PwP) are also susceptible to psychiatric symptoms such as depression, hallucinations, and confusion, as well as the likelihood of falls and freezing of gait as the condition progresses [7]. To date, the impact of maintaining a caregiving role for people with PwP has been largely restricted to the assessment of caregiver burden and caregiver strain. Previous studies suggest that increased burden and strain are associated with the duration of caring, the physical health of the patient, including their increasing disability and propensity to falls, patient psychiatric symptoms, including behavioural disturbances (such as impulse control disorders and apathy), the age of the carer, and carer mood [8–16].

Although the concepts of caregiver burden and strain have not been well defined in the literature, they suggest a direct measure of the duty of caring. This is in contrast to the concept of quality of life (QoL) which assesses a far broader spectrum relating to an individual's overall well being [17]. The few studies that have assessed QoL in carers of PwP suggest that their QoL is related to the age and the functional state of the PwP, duration of PD, household income, and carer mood [18–20]. These reported associations are similar to those reported in relation to caregiver burden and caregiver strain; however, such studies have been limited by their small sample sizes and the types of measures that were used, that is, generic QoL measures or poorly validated disease specific tools.

Given this background, this study aims to make an assessment of factors influencing the QoL of carers and subsequently consider results in the context of current clinical guidelines for the management of Parkinson's. The recent development and validation of a PD specific carer QoL measure, the PDQ-Carer [17], has been a significant step forward towards achieving this aim as such a tool is likely to be more sensitive than generic QoL measures used in previous studies [18–20]. 

## 2. Methods

Ethical approval for the study was granted by the Central University Research Ethics Committee and the Medical Sciences Interdivisional Research Ethics Committee of the University of Oxford.

### 2.1. Participants

PwP and their carers were invited to take part in a postal survey via three routes. Firstly, branch and volunteer support officers from Parkinson's UK (a support and research charity for PD) were asked to promote the study to their members. Secondly, 26 local branches of Parkinson's UK sent information about the study to convey to their members. Thirdly an advertisement was placed on the Parkinson's UK Research Opportunity website.

### 2.2. Measures

Carers completed the PDQ-Carer, a 29-item instrument answered on a 5-point Likert scale [17]. The questionnaire contains four domains (Social and Personal Activities, Anxiety and Depression, Self-Care, and Stress) and has been shown to have good psychometric properties both in terms of internal consistency reliability and construct validity [17]. Raw scores for each domain are transformed to have a range of 0 to 100, with lower scores indicating superior QoL. PwP completed the Parkinson's Disease Quality of Life Questionnaire (PDQ-39), a 39-item instrument answered on a 5-point Likert scale [21]. The questionnaire contains eight domains (Mobility, Activities of Daily Living, Emotional Well Being, Stigma, Social Support, Cognitions, Communication, and Bodily Discomfort) and is regarded as “the most thoroughly tested and applied questionnaire” for assessment of QoL in PD [22]. Raw scores for each domain are transformed to have a range from 0 to 100 with lower scores indicating superior QoL. Questionnaires were administered by post. Reminder letters were sent four weeks after the original mailing.

### 2.3. Statistical Analysis

Data were checked for the presence of outliers and normality of distribution prior to statistical analysis. Independent samples *t*-tests were calculated to assess differences between groups (gender and health status). Stepwise multiple regression analyses were conducted to assess predictors of carer quality of life. Domains from the PDQ-39 that were highly skewed or that demonstrated significant collinearity with others were omitted. Data were analysed using SPSS Version 20.

## 3. Results

A response rate of 61% was achieved. Sample characteristics for both carers and PwP are given in Table 1. Carers were predominantly female, with over half of respondents reporting that they themselves had a chronic illness or condition. The vast majority, 92%, identified themselves as a spouse or partner.

Comparisons by gender (Table 2) identify female carers as reporting significantly inferior QoL as measured by all four domains of the PDQ-carer when compared to male caregivers: Social and Personal Activities, *t* = −2.76, *P* < 0.01; Anxiety and Depression; *t* = −2.94, *P* < 0.01; Self-Care, *t* = −3.71, *P* < 0.001; Stress, *t* = −4.01, *P* < 0.001.

Comparisons by carer health status (presence or absence of a long-term condition or illness) are summarised in Table 3. Caregivers with a long-term condition report significantly inferior QoL in all of the four domains of the PDQ-carer when compared to healthy carers: Social and Personal Activities, *t* = 2.50, *P* < 0.01; Anxiety and Depression; *t* = 3.54, *P* < 0.001; Self-Care, *t* = 3.30, *P* < 0.001; Stress, *t* = 3.08, *P* < 0.01.

Analyses to identify factors implicated in these wide differences in carer QoL by health status assessed carer age, duration of caregiving, and age and QoL of the PwP as measured by the PDQ-39. A significant difference was identified by the age of carer, with those experiencing a long-term condition being significantly older than healthy carers (*t* = 3.76, *P* < 0.001). No significant differences were found in any of the other variables assessed.

### 3.1. Predictors of Carer QoL

Stepwise multiple regression analyses were performed to identify predictors of the four domains of carer quality of life. Variables included carer age, length in the caregiving role, and PwP QoL as measured by the PDQ-39. The PDQ-39 domains of Stigma and Social Support were removed due to being highly skewed (i.e., low/superior QoL scores). The Activities of Daily Living domain was also removed due to collinearity with the Mobility domain. Results of the analyses are summarised in Tables 4(a)–4(d).

Analysis of carer Social and Personal Activities (Table 4(a)) identified four predictors explaining 35.4% of the variance (*R* ^2^ = .37, *F*(4,203) = 29.37, *P* < 0.001). Significant predictors included PwP mobility (*β* = .35, *P* < 0.001), PwP cognitive impairment (*β* = .26, *P* < 0.001), carer age (*β* = .20, *P* < 0.01), and duration of caregiving (*β* = .12, *P* < 0.05).

Analysis of carer Anxiety and Depression (Table 4(b)) identified four predictors explaining 28.6% of the variance (*R* ^2^ = .30, *F*(4,200) = 21.41, *P* < 0.001). Significant predictors included PwP cognitive impairment (*β* = .33, *P* < 0.001), PwP mobility (*β* = −.19, *P* < 0.01), carer age (*β* = .18, *P* < 0.01), and duration of caregiving (*β* = .16, *P* < 0.01).

Analysis of carer Self-Care (Table 4(c)) identified three predictors explaining 28.3% of the variance (*R* ^2^ = .29, *F*(3,205) = 28.31, *P* < 0.001). Significant predictors included PwP mobility (*β* = .33, *P* < 0.001), PwP cognitive impairment (*β* = −.25, *P* < 0.001), and duration of caregiving (*β* = .15, *P* < 0.01).

Analysis of carer Stress (Table 4(d)) identified four predictors explaining 26.7% of the variance (*R* ^2^ = .28, *F*(4,203) = 19.88, *P* < 0.001). Significant predictors included PwP cognitive impairment (*β* = .34, *P* < 0.001), duration of caregiving (*β* = −.18, *P* < 0.01), PwP mobility (*β* = .17, *P* < 0.01), and carer age (*β* = .13, *P* < 0.05).

## 4. Discussion

The current study has aimed to make an assessment of factors influencing the QoL of carers of PwP. Achieving this aim has been significantly aided by the development and validation of a PD specific carer QoL questionnaire [17]. The PDQ-Carer is a short meaningful measure which taps areas of particular salience and concern to carers of PwP. As such, it is likely to be more sensitive than generic QoL measures and thereby lead to a greater understanding of the factors influencing QoL in this group.

The findings reported identify a number of factors influencing carer QoL. Analyses demonstrated that female carers report significantly inferior QoL in comparison to male carers. Although such gender differences are not uncommon in QoL data [23], differences in the PD caregiver burden and strain literature are inconsistent, with some demonstrating significant differences [24, 25] and others failing to do so [14, 26]. However, of the few studies that have specifically assessed caregiver QoL in PD [18–20], none has included gender of the carer in their analyses. Differences by carer gender have previously been explained as a function of the coping strategies employed by males and females. More specifically, it is suggested that the problem focused coping generally incorporated by males is a more effective strategy than the emotion focused coping utilised by females [27]. Regardless of the underlying mechanism, the significant findings reported here may have practical relevance similar to that identified in the literature relating to carers of Alzheimer's patients, where it has been recommended that nurses should place more emphasis on helping female carers [28].

Results that identify carers with a long-term condition themselves experiencing significantly inferior QoL when compared to healthy carers appear intuitive. In an attempt to explore this further, an assessment was made as to whether carer age, duration of caregiving, and PwP age, and QoL might be implicated. The finding that only the age of the carer significantly differed between healthy carers and those carers experiencing a long-term condition may again have practical relevance, with greater emphasis needing to be placed on older carers by practitioners.

Regression analyses further strengthen the findings reported previously. PwP mobility and cognitive impairment appear to exert significant influences on caregiver QoL as demonstrated in previous studies [11, 13, 16, 18, 29]. Additionally, results suggest that duration of caring and carer age are significant factors, with older carers experiencing inferior QoL. Previous studies have also identified increasing age as a significant factor [13, 30, 31], and the current study suggests that this is particularly evident in relation to engagement in social and personal activities and mental health. The importance of maintaining an active social and personal life with regard to healthy ageing is well documented [32], and maintenance of this should be of significant concern in the caregiver population. Additionally, declining mental health is highly prevalent in the older population [33, 34], and it is likely that this could be further affected by a caregiving role, something that requires close monitoring by relevant professionals.

The results reported have policy relevance, and none more so than for clinical guidance in the management of PD. Current UK clinical guidelines [35] place little emphasis on the carers of PwP. Guidance that is given focuses almost entirely on communication and information. The guidelines suggest carers require general information about PD, specific information about the person with PD (if permission is given), details of services, advice regarding effective communication as PD progresses, as well as information and support to maintain their own health and well being.

The data presented from this study suggest far broader guidance is required when considering carers of PwP and that there are multiple influences on their QoL. PwP levels of mobility and cognitive impairment are significant influences on carer QoL. Carer age and length of time in the caregiving role are also of importance, and females appear more vulnerable in their caregiving role than males do. Not only should practitioners and service providers be aware of the heightened impact of PD on carers over time and as PwP symptoms deteriorate, but this should also be reflected in guidance for the management of PD. Further studies, possibly of a qualitative nature, should be conducted to identify the specific needs of those who fall into the more vulnerable groups identified in order to facilitate the inclusion of such guidance. 

A number of shortcomings from the reported study need to be acknowledged. It may be that members of a PD support group are not representative of all informal PD carers. They may be a specific sample of carers who have sought membership in a support group due to particular circumstances. This clearly has the potential to introduce an element of bias into the results. Additionally, it is acknowledged that this study assessed only PwP perceptions of their QoL, and that further studies focusing specifically on objective and clinical disorder aspects as they relate to PD carer QoL would further enhance the literature. Finally, there is a limit to what can be concluded from a cross-sectional design such as that employed in this study. Longitudinal data is required to make a more detailed evaluation of the well being of carers of PwP over time.

## 5. Conclusions

Despite recognition from the UK government regarding the contribution of carers, their needs are not sufficiently recognised in current clinical guidelines for PD. Data from this and other studies suggest that this should be addressed, and further research may allow for more specific and detailed recommendations to be proposed.

## Figures and Tables

**Table 1:** Sample characteristics of carers and people with Parkinson's.

	Caregivers	People with Parkinson's
	*N* = 238	*N* = 238
Gender M : F	60 : 177	169 : 66
Mean age (years)	68.2 (SD 9.48, range 25–89)	71.6 (SD 7.78, range 44–87)
Mean length of caregiving role (years)	8.1 (SD 7.46, range 1–51)	
Has long-term condition Y : N	122 : 111	

**Table 2:** Comparisons of carer quality of life by gender.

PDQ-Carer domain	Male carers Mean (SD)	Female carers Mean (SD)	*t*	*P*
Social and Personal Activities	38.12 (27.81)	49.02 (25.87)	−2.76	<0.01
Anxiety and Depression	38.41 (25.47)	49.30 (24.21)	−2.94	<0.01
Self-Care	22.67 (23.83)	36.05 (24.19)	−3.71	<0.001
Stress	34.18 (24.41)	48.27 (22.98)	−4.01	<0.001

**Table 3:** Comparisons of carer quality of life by health status.

PDQ-Carer domain	Carer has long-term condition Mean (SD)	Carer does not have long-term condition Mean (SD)	*t*	*P*
Social and Personal Activities	50.51 (26.70)	41.78 (26.33)	2.50	<0.01
Anxiety and Depression	52.20 (25.92)	40.79 (22.52)	3.54	<0.001
Self-Care	38.02 (25.25)	27.48 (23.15)	3.30	<0.001
Stress	49.45 (25.02)	39.83 (22.28)	3.08	<0.01

**Table:** (a)

Carer Social and Personal Activities	*β*	*R*	*R* ^2^	*R* ^2^ change	Adjusted *R* ^2^
PwP mobility	.35	.51	.26	.26	.26
PwP cognitive impairment	.26	.56	.31	.05	.31
Carer age	.20	.59	.35	.04	.34
Duration of caring	.12	.60	.37	.02	.35

**Table:** (b)

Carer Anxiety and Depression	*β*	*R*	*R* ^2^	*R* ^2^ change	Adjusted *R* ^2^
PwP cognitive impairment	.33	.44	.19	.19	.19
PwP mobility	.19	.49	.24	.05	.23
Carer age	.18	.52	.27	.03	.26
Duration of caring	.16	.55	.30	.03	.29

**Table:** (c)

Carer Self-Care	*β*	*R*	*R* ^2^	*R* ^2^ change	Adjusted *R* ^2^
PwP mobility	.33	.47	.22	.22	.22
PwP cognitive impairment	.25	.52	.27	.05	.26
Duration of caring	.15	.54	.29	.02	.28

**Table:** (d)

Carer Stress	*β*	*R*	*R* ^2^	*R* ^2^ change	Adjusted *R* ^2^
PwP cognitive impairment	.34	.44	.20	.20	.19
Duration of caring	.18	.49	.24	.04	.23
PwP mobility	.17	.51	.26	.02	.25
Carer age	.13	.53	.28	.02	.27
